# Transient cellular adhesion on poly(ethylene-glycol)-dimethacrylate hydrogels facilitates a novel stem cell bandage approach

**DOI:** 10.1371/journal.pone.0202825

**Published:** 2018-08-23

**Authors:** Rosita R. Asawa, Jessica C. Belkowski, Daniel A. Schmitt, Elizabeth M. Hernandez, Ann E. Babcock, Christina K. Lochner, Holly N. Baca, Colleen M. Rylatt, Isaac S. Steffes, Jace J. VanSteenburg, Karina E. Diaz, Derek M. Doroski

**Affiliations:** Department of Biology, Franciscan University of Steubenville, Steubenville, OH, United States of America; Kyoto Daigaku, JAPAN

## Abstract

We discovered a transient adhesion property in poly(ethylene glycol) dimethacrylate (PEG-DMA) hydrogels and employed it to develop a novel “stem cell bandage” model of cellular delivery. First, we cultured human mesenchymal stromal cells (MSCs) on the surface of PEG-DMA hydrogels with high amounts of arginine-glycine-aspartic acid (RGD) adhesive peptides (RGD++) or without RGD (RGD-). On day 1, MSCs underwent an initial adhesion to RGD- hydrogels that was not significantly different over 13 days (n = 6). In addition, cells appeared to be well spread by day 3. Significantly fewer cells were present on RGD- hydrogels on day 15 compared to day 9, suggesting that RGD- hydrogels allow for an initial cellular adhesion that is stable for multiple days, but transient over longer periods with a decrease by day 15. This initial adhesion is especially surprising considering that PEG-DMA does not contain any biological adhesion motifs and is almost chemically identical to poly(ethylene glycol) diacrylate (PEG-DA), which has been shown to be non-adhesive without RGD. We hypothesized that MSCs could be cultured on RGD- PEG-DMA hydrogels and then applied to a wound site to deliver cells in a novel approach that we refer to as a “stem cell bandage”. RGD- donor hydrogels were successfully able to deliver MSCs to PEG-DMA acceptor hydrogels with high RGD content (RGD++) or low amounts of RGD (RGD+). Our novel “bandage” approach promoted cell delivery to these model surfaces while preventing cells from diffusing away. This stem cell delivery strategy may provide advantages over more common stem cell delivery approaches such as direct injections or encapsulation and thus may be valuable as an alternative tissue engineering approach.

## Introduction

Numerous tissues have been targeted in tissue engineering approaches including cartilage [1], skin, bone [2], teeth [3], blood vessels [4], and intestine [5]. Mesenchymal stromal cells (MSCs) are commonly employed in tissue engineering. MSCs are multipotent progenitor cells with the capacity to differentiate down multiple lineage lines including fibroblasts, osteocytes, chondrocytes, and adipocytes [6]. In addition to their differentiation potential, MSCs secrete relatively high levels of growth factors, inhibit scarring, promote angiogenesis, and release immunomodulatory chemicals that allow these cells to be used allogenically [6]. In addition to ease of growth and expansion *in vitro*, MSCs may be isolated and reintroduced to the patient of interest, reducing the danger of immunologic rejection [7]. Tissue engineering strategies often involve combining MSCs with biomaterials to promote differentiation or stimulate other beneficial MSC behaviors.

Poly(ethylene-glycol) (PEG)-based polymers are commonly used as biomaterials for tissue engineering. PEG has many favorable properties, such as biocompatibility and biodegradability, making it ideal for insertion into the body. However, PEG by itself is relatively biologically inert and requires modification to become bioactive and allow for cell adhesion and proliferation on its surface [1]. Arg-Gly-Asp (RGD), an adhesive peptide found in the cell attachment region of fibronectin, is one of the most common modifications for hydrogels [8]. Several derivatives of PEG have been investigated for use as biomaterials, including poly(ethylene-glycol)-diacrylate (PEG-DA) and poly(ethylene-glycol)-dimethacrylate (PEG-DMA). These nearly identical polymers are used for scaffolding purposes due to their biocompatibility, which allows for cell viability up to several weeks [9,10]. In previous studies, RGD adhesive peptides have been incorporated into PEG-DA hydrogels to promote cellular adhesion and spreading that is not possible in the absence of the RGD peptides [11]. Cells have also been shown to adhere to PEG-DMA hydrogels modified with RGD [12].

Functions of hydrogel biomaterials span a wide range of applications including drug delivery, neural tissue engineering, and cancer research [13–15]. One common tissue engineering approach is to add MSCs to an injectable PEG-hydrogel that rapidly crosslinks when placed in the body at the wound site. This approach results in MSCs that are encapsulated inside the hydrogel, effectively trapping the MSCs in one area [16]. Other approaches involve injection of MSCs directly to the wound site [17] or systemic administration via intravenous (IV) injection or intra-arterial (IA) injection [18].

However, current cell delivery models are not without their drawbacks. MSCs directly injected into the body without a biomaterial matrix may not remain at the target site. For example, MSC delivery via both systemic and local administration has resulted in cells accumulating in areas other than the targeted wound site [18]. In contrast, MSCs encapsulated in hydrogels may constrain the cells to the site of interest, but the majority of the MSCs remain trapped within the hydrogel and cannot be in direct contact with the wound. While the encapsulated MSCs would still be able to secrete growth factors and immunomodulatory molecules, cell proliferation is hindered, potentially reducing the effectiveness of the therapy [19]. This study reveals unexpected transient adhesion properties of unmodified PEG-DMA hydrogels and explores the usage of these properties for a novel approach to stem cell delivery. In our approach MSCs are seeded on the surface of unmodified PEG-DMA hydrogels and placed in direct contact with the desired area, creating a “bandage”-like delivery model. This approach may overcome some limitations of other approaches by trapping stem cells at the target site while still allowing cellular proliferation and access to the wound.

## Results

### Trilineage differentiation potential

**Fig 1:**
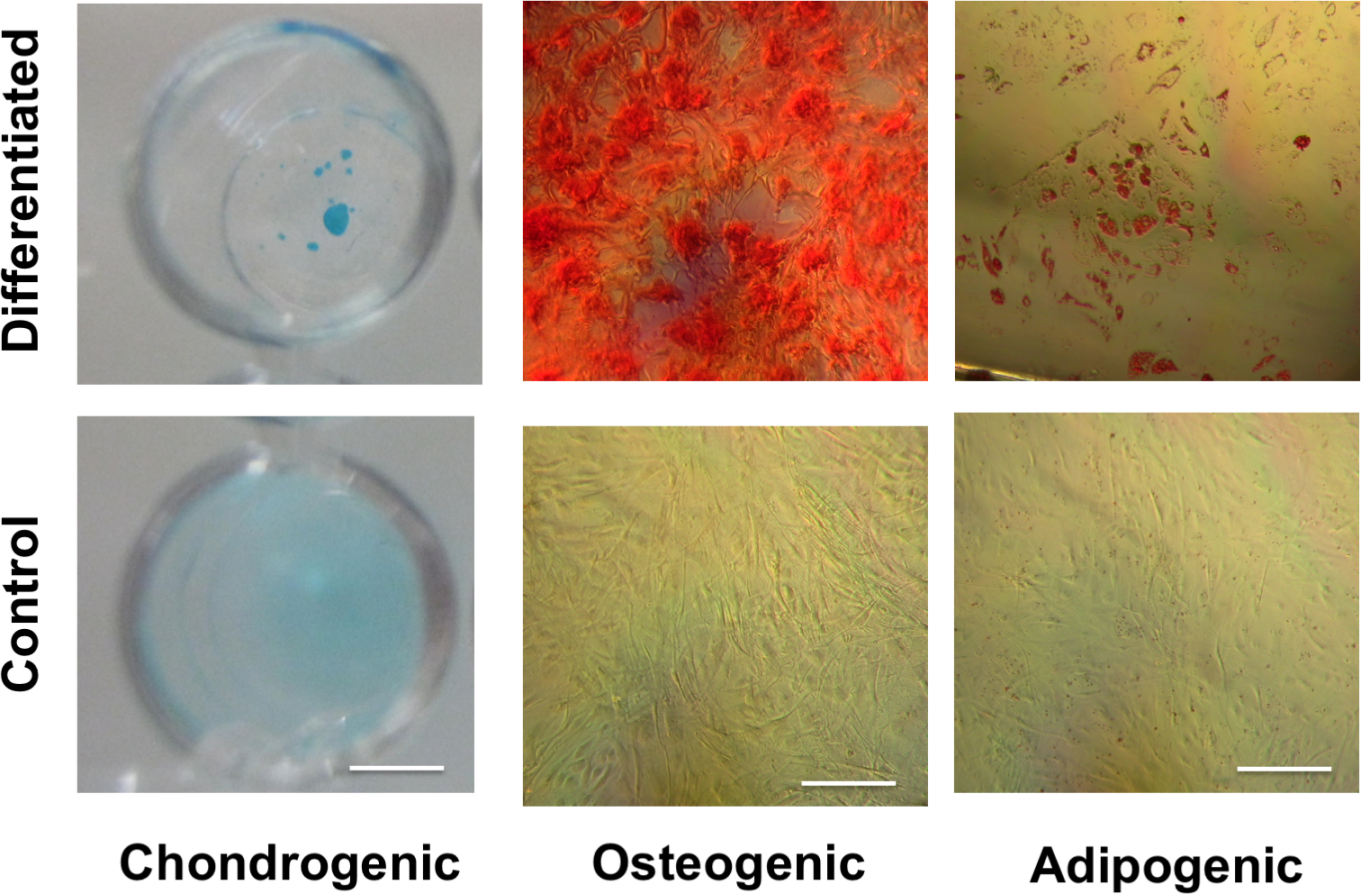
MSCs demonstrate trilineage differentiation potential. Staining of cells cultured in chondrogenic, osteogenic, or adipogenic differentiation medium (top row) showed production of molecules associated with chondrogenic (glycosaminoglycans, dark blue), osteogenic (calcium deposits, red), or adipogenic (lipids, red) differentiation. Staining of cells cultured in basal medium (bottom row) did not show evidence of differentiation. The scale bar in the chondrogenic images is 6.5 mm. The scale bar in the osteogenic and adipogenic images is 40 μm.

MSC pellets in chondrogenic differentiation medium for 21 days produced a dark blue color under Alcian Blue staining, indicating the presence of glycosamioglycans (Fig 1). In contrast, MSCs cultured in basal medium had a light blue color under Alcian Blue, indicating a lack of glycosaminoglycan production. MSCs cultured in osteogenic differentiation medium produced a dark red color under Alizarin Red staining, indicating the presence of calcium deposits (Fig 1). In contrast, MSCs cultured in basal medium showed no evidence of red coloration under Alizarin Red, indicating a lack of calcium production. MSCs cultured in adipogenic medium for 21 days produced areas of red staining under Oil Red O, indicating the presence of lipids (Fig 1). In contrast, MSCs cultured in basal medium showed little evidence of accumulation of red coloration under Oil Red O, indicating a lack of lipid accumulation.

### PEG-DA cellular attachment study

**Fig 2:**
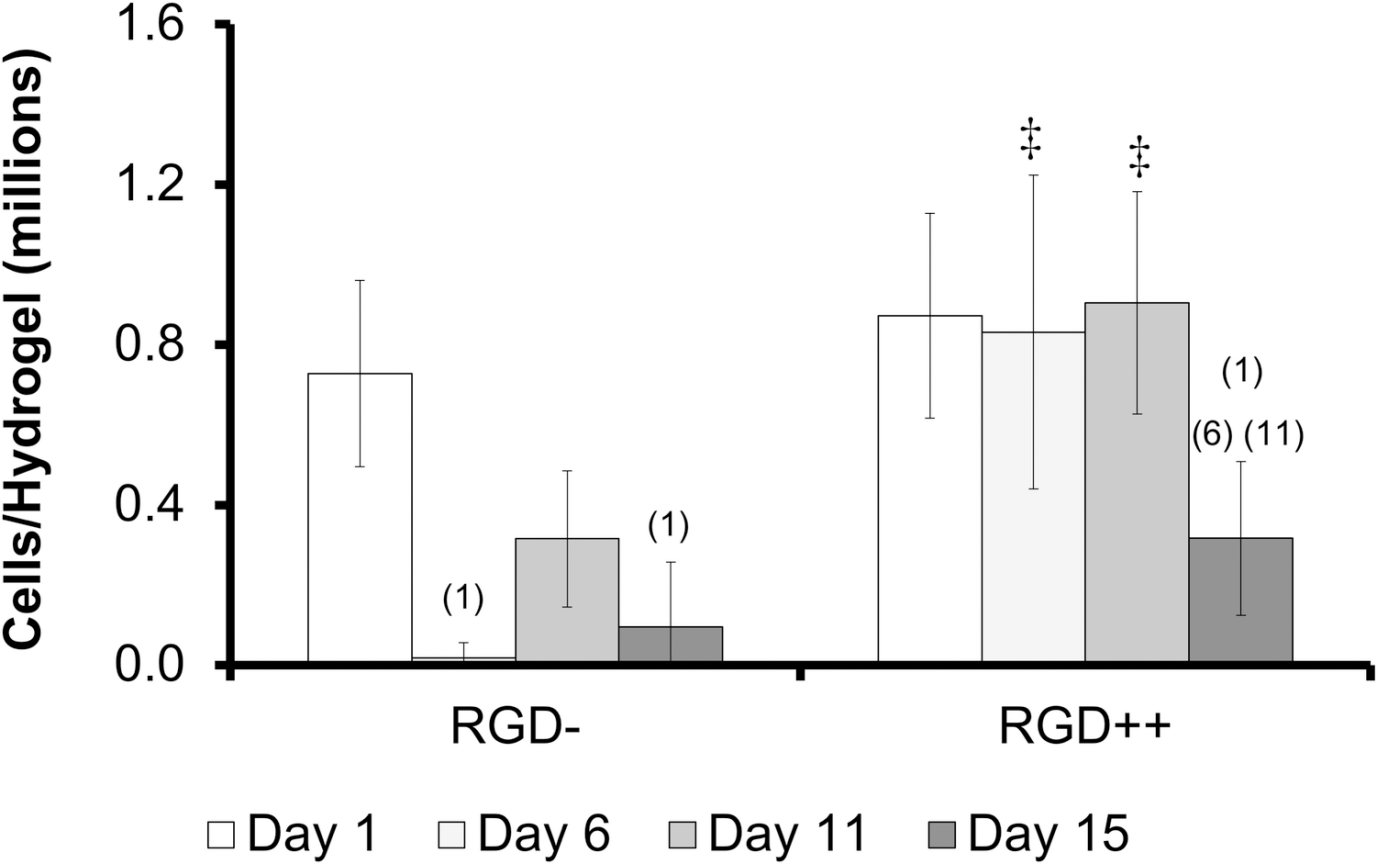
Cells adhere over time to PEG-DA hydrogels with RGD (RGD++), but not without RGD (RGD-). Number of cells present on RGD- and RGD++ hydrogels after 1, 6, 11, and 15 days (n = 6 ± standard deviation). ^(1)^Significance vs. day 1 for the same sample type. ^(6)^Significance vs. day 6 for the same sample type. ^(11)^Significance vs. day 11 for the same sample type. ^‡^significance from RGD- hydrogels at same time point (p < 0.05).

**Fig 3:**
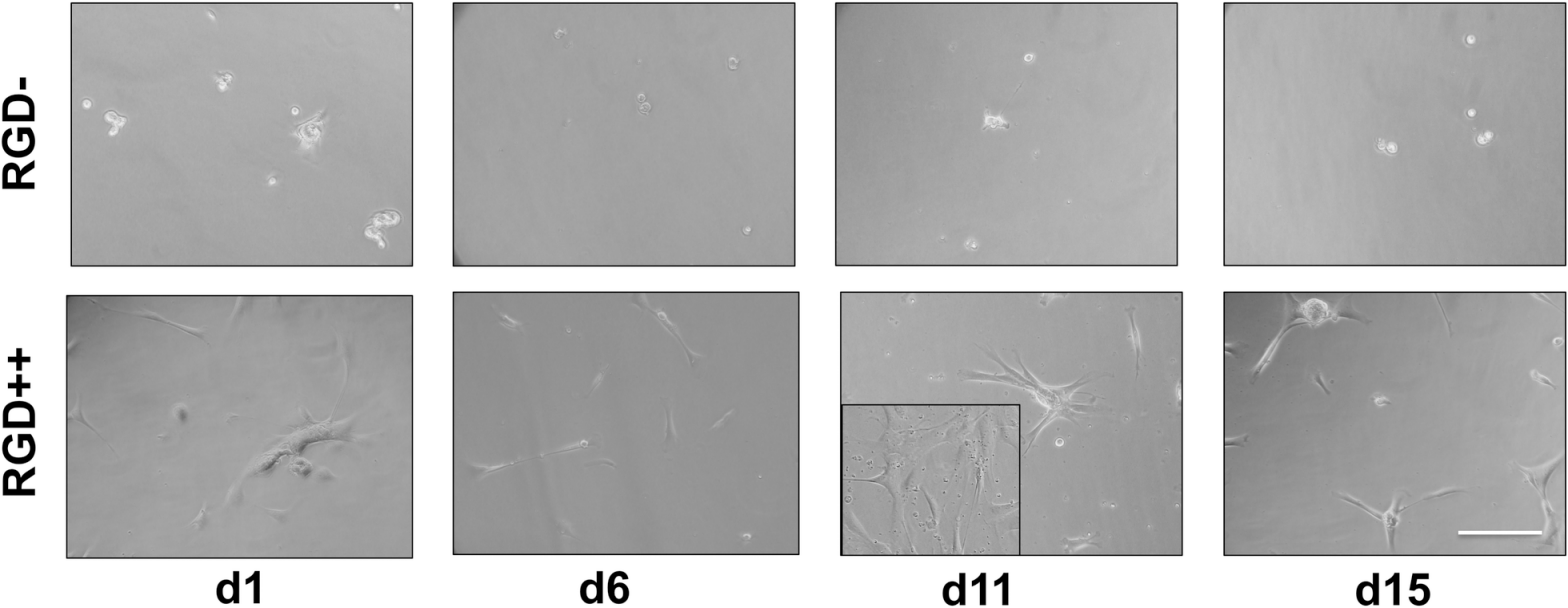
Cellular adhesion on PEG-DA hydrogels. Cells adhered to PEG-DMA hydrogels with RGD peptides (RGD++) on days 1, 6, 11, and 15. Cells appeared more rounded and sparse on hydrogels without RGD (RGD-). Inset: an alternate area of the hydrogel showing a different pattern of cellular adhesion. Scale bar = 10 μm.

The number of cells collected from RGD- hydrogels was significantly lower on day 6 and day 15 compared to day 1 (Fig 2). The number of cells on RGD++ hydrogels was significantly higher on day 1, 6, and 11 compared to day 15 (Fig 2). On days 6 and 11 the RGD++ hydrogels had significantly more cells present than RGD- hydrogels. The quantitative data generally matched well with the images (Fig 3). However, it can be difficult to draw general trends from the images alone as the distribution of cells on the hydrogels did not always appear uniform. For example, on day 11 some areas of the RGD++ hydrogel appeared to have a moderate cellular density and other areas appeared to have a high cellular density (Fig 3 inset). In addition, cells found on RGD- PEG-DA hydrogels generally appeared to be more rounded while spreading of cells was more apparent on RGD++ hydrogels. When observing patterns of cellular adhesion on hydrogels in this study and the other studies in this work, the researchers noted that the cellular density often appeared greater toward the center of the hydrogel compared to the edges.

### PEG-DMA cellular release study

**Fig 4:**
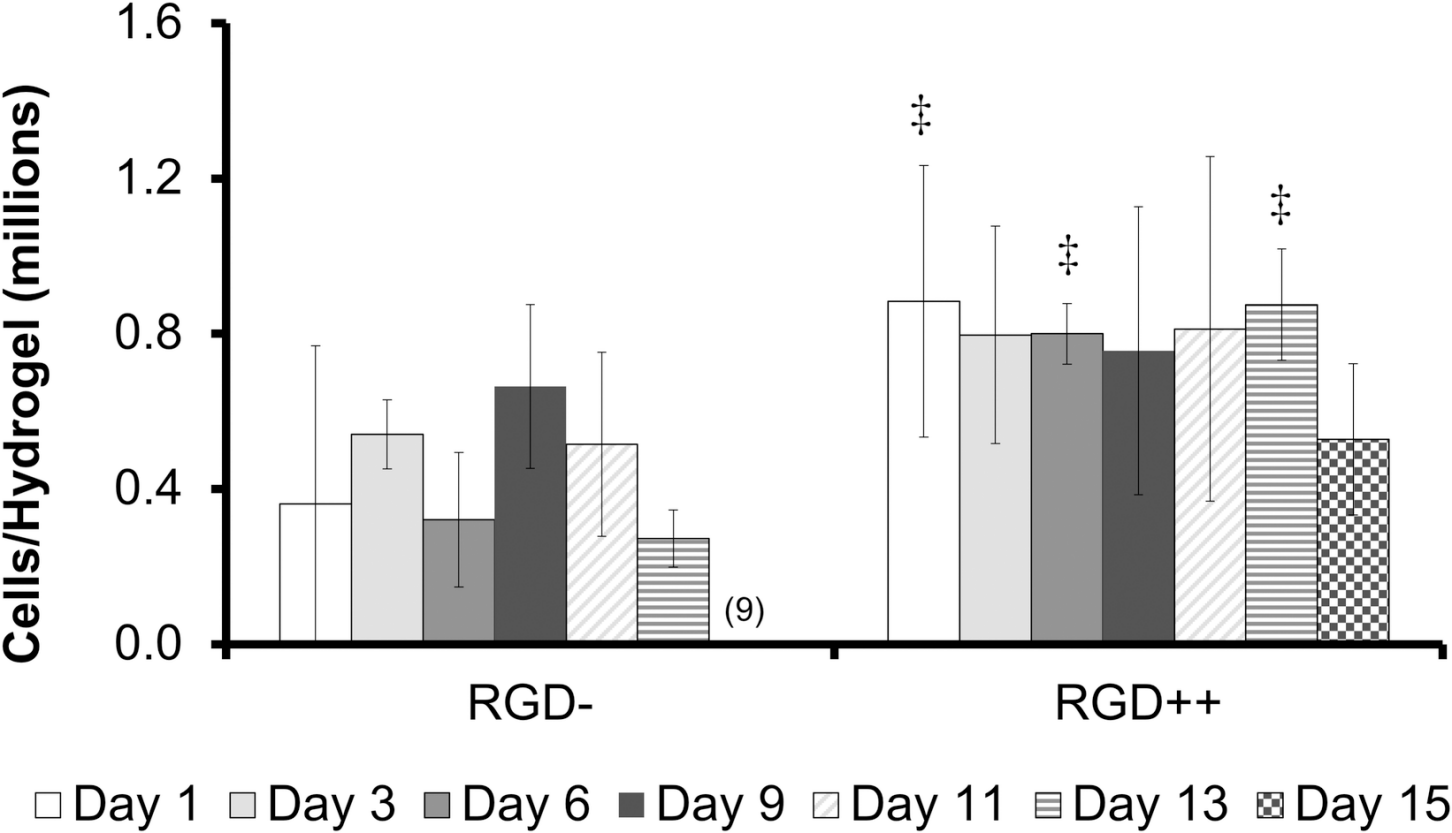
Timeline of MSC adhesion varies on PEG-DMA hydrogels with RGD (RGD++) or without RGD (RGD-). Number of MSCs present on RGD- and RGD++ hydrogels after 1, 3, 6, 9, 11, 13, and 15 days (n = 6 ± standard deviation). ^(9)^Significance vs. day 9 for the same sample type, ^‡^significance from RGD- hydrogels at same time point (p < 0.05).

**Fig 5:**
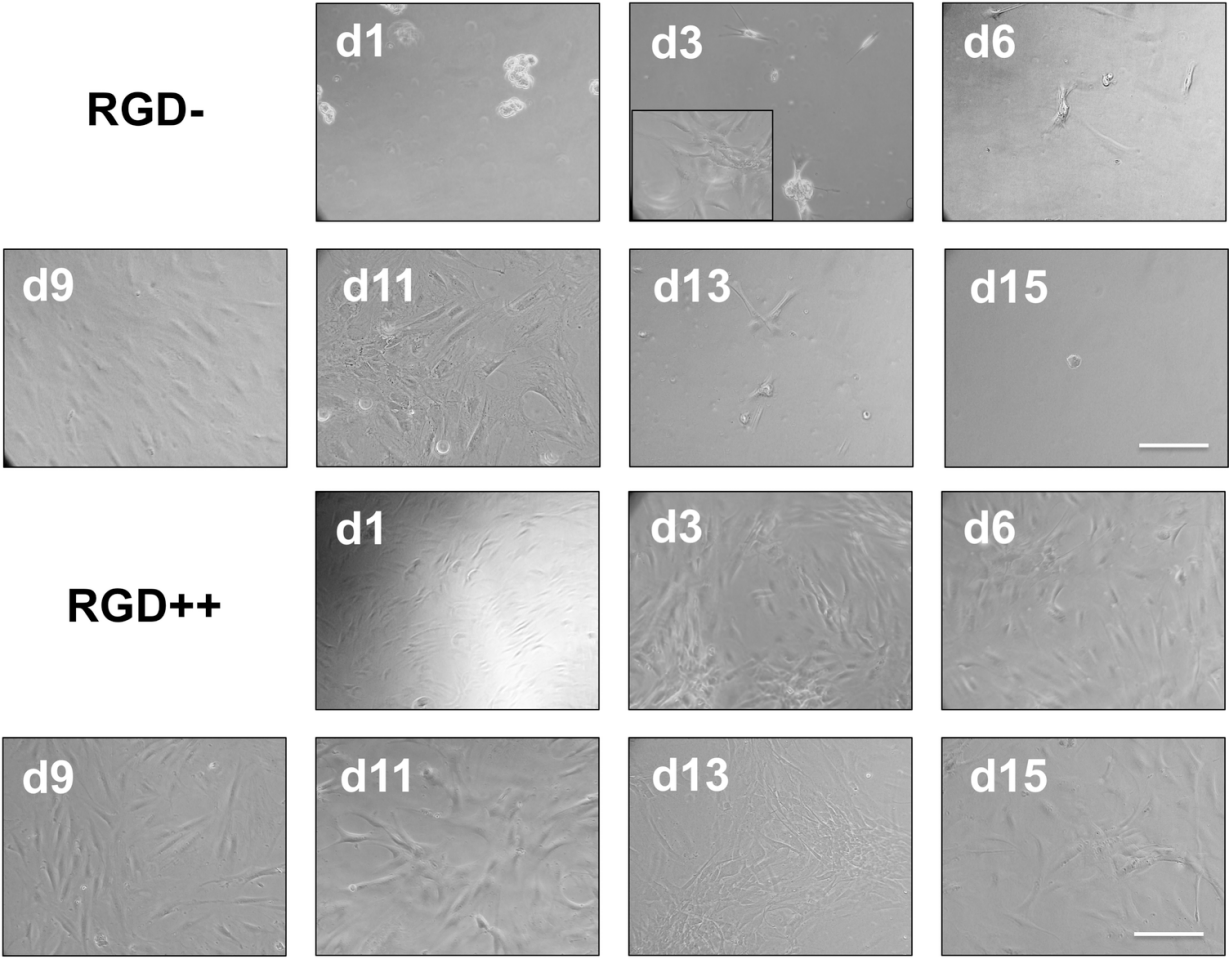
MSC adhesion on PEG-DMA hydrogels. MSCs adhered to PEG-DMA hydrogels on days 1, 3, 6, 9, 11, 13, and 15. Top two rows: hydrogels without RGD peptides (RGD-). Bottom two rows: hydrogels with RGD peptides (RGD++). Inset: an alternate area of the d3 RGD- hydrogel showing a different pattern of cellular adhesion. Scale bar = 10 μm.

The number of MSCs collected from RGD- hydrogels was significantly higher on day 9 compared to day 15 (Fig 4). Similarly, a repeated version of this experiment with time points on only day 1, day 11, and day 14 showed a significantly higher number of cells on day 11 compared to day 1, but this significance disappeared on day 14 (S1 Fig). The number of cells on RGD++ hydrogels was not significantly different over time (Fig 4). On days 1, 6, and 13 the RGD++ hydrogels had significantly more cells present than RGD- hydrogels. The quantitative data generally matched well with the images (Fig 5 and S2 Fig). However, it can be difficult to draw general trends from the images alone as the distribution of cells on the hydrogels did not always appear uniform. For example, on day 3 RGD- hydrogels, some areas of the hydrogels appeared to have a moderate cellular density and other areas appeared to have a much higher number of cells (Fig 5 inset).

### Delivery to strong acceptor hydrogels

**Fig 6:**
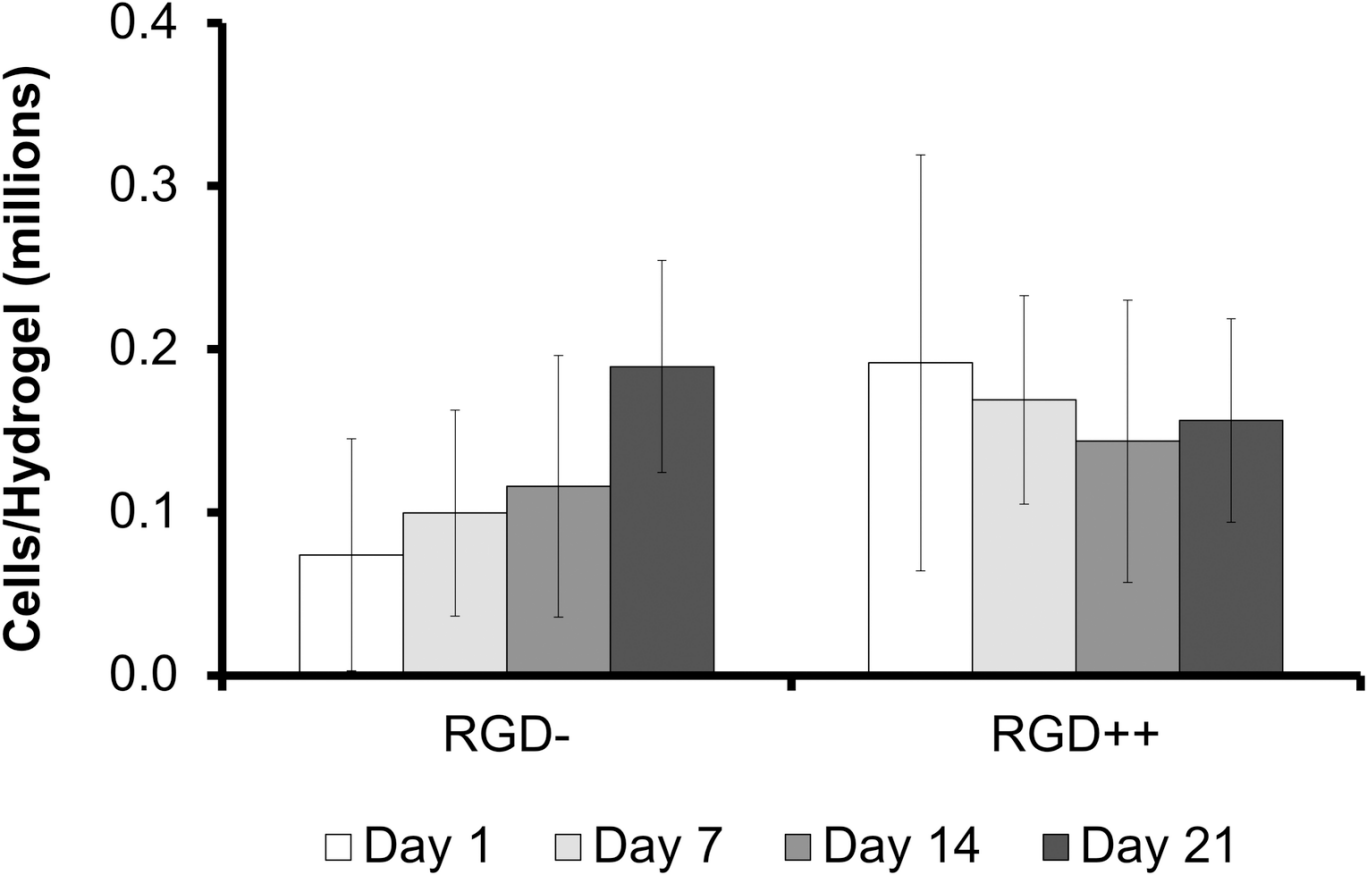
MSCs delivered to RGD++ hydrogels (strong acceptors). Cells were successfully delivered from RGD- donor hydrogels (strong donor) and RGD++ donor hydrogels (weak donors) to the RGD++ acceptor hydrogel surface over time. Cell numbers on RGD++ acceptor hydrogels were measured after 1, 7, 14, and 21 days (n = 6 ± standard deviation). There were no significant differences between hydrogels of any type at p < 0.05.

**Fig 7:**
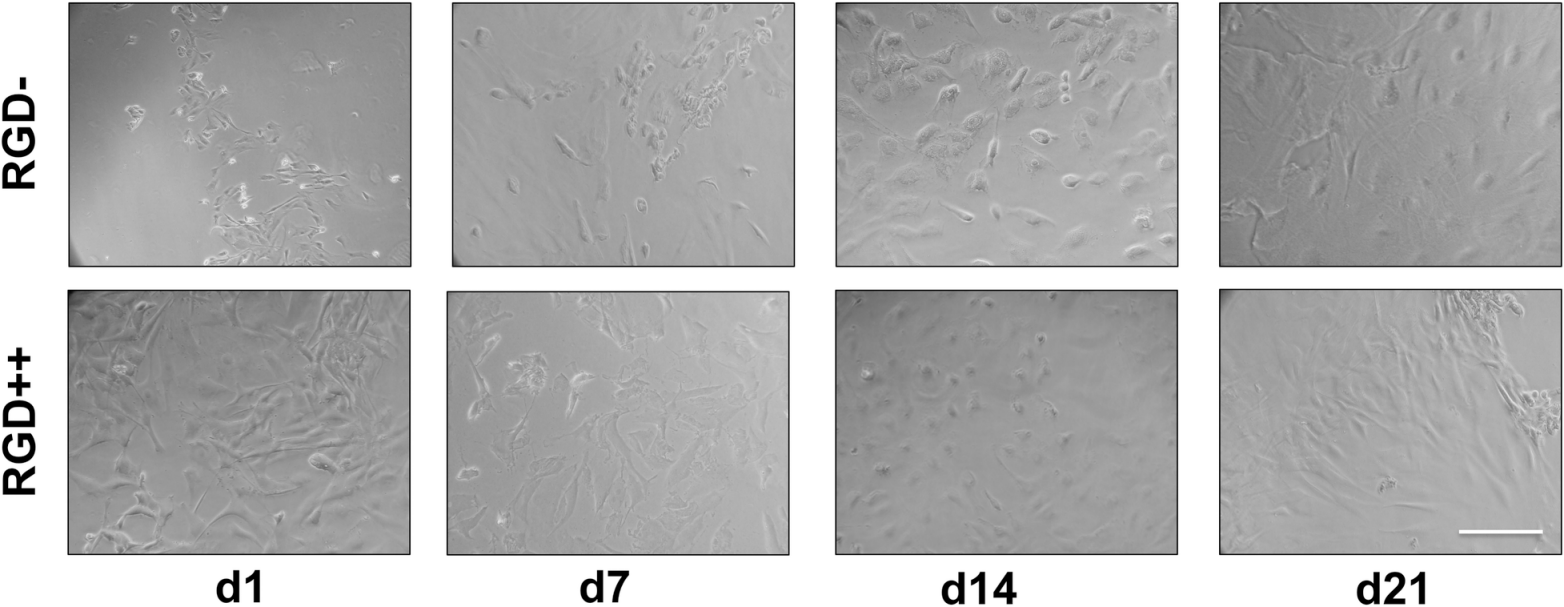
Cells delivered to RGD++ hydrogels (strong acceptors). Cells were delivered to RGD++ PEG-DMA hydrogels on days 1, 7, 14, and 21 from both RGD- donor hydrogels (strong donors) and RGD++ donor hydrogels (weak donors). Scale bar = 10 μm.

The number of MSCs delivered from RGD- donor hydrogels to the RGD++ acceptor hydrogels was not significantly different over time (Fig 6). There was also no significant difference in the number of cells delivered by the RGD++ donor hydrogels to RGD++ acceptor hydrogels over time or between the cells delivered by RGD++ donor hydrogels compared to RGD- donor hydrogels. Results generally matched well with images (Fig 7). Similar results were obtain in a second run of the experiment (S3 Fig).

### Delivery to weak acceptor hydrogels

**Fig 8:**
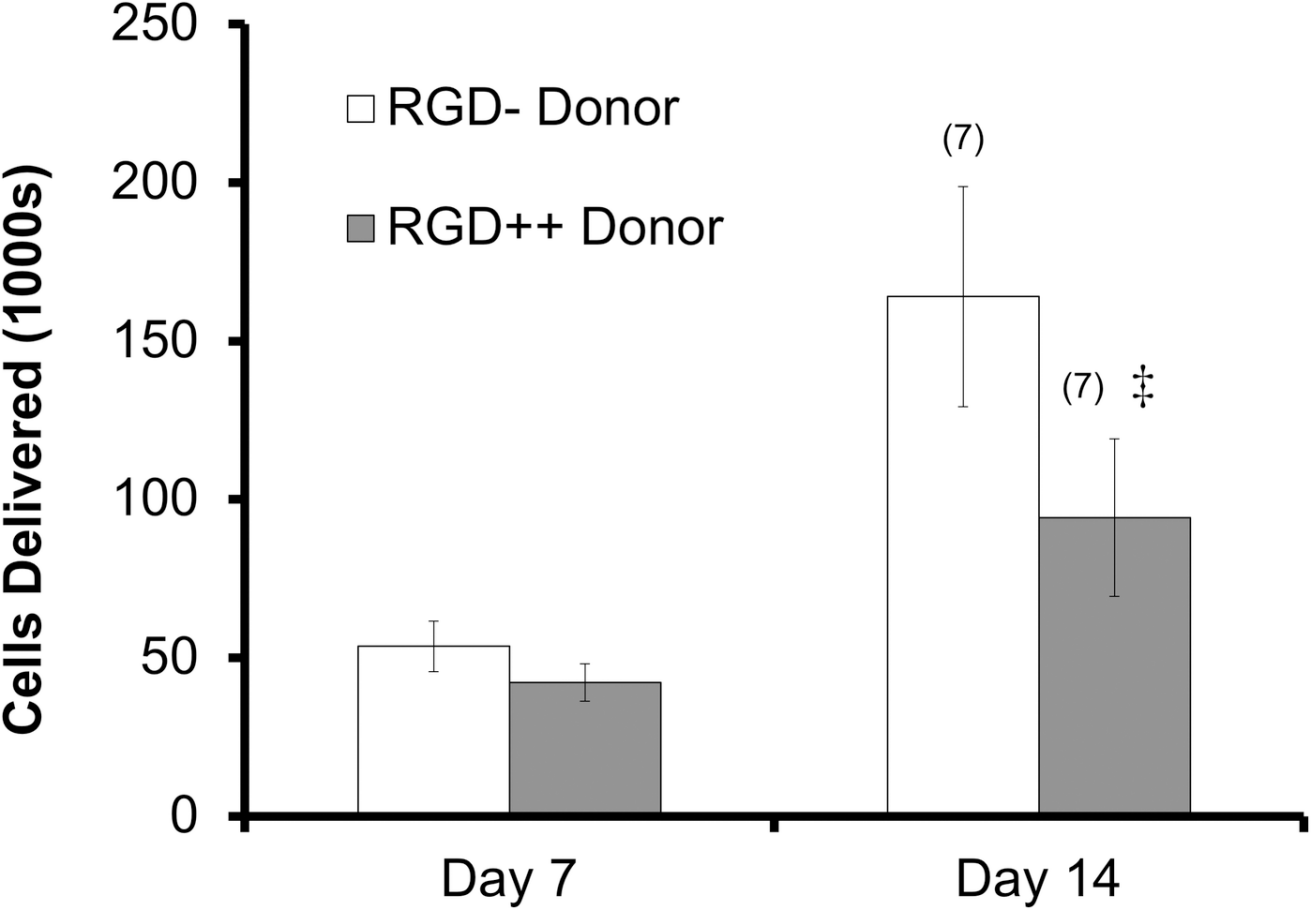
MSCs delivered to RGD+ hydrogels (weak acceptors). MSCs were delivered to RGD+ acceptor hydrogel surfaces after 7 and 14 days (n = 5 ± standard deviation). ^(7)^Significance vs. day 1 for the same sample type. ^‡^significance from RGD- at the same time point (p<0.05).

The number of MSCs delivered to RGD+ acceptor hydrogels was significantly higher on day 14 compared to day 7 for both RGD- donor and RGD++ donor hydrogels (Fig 8). The number of cells delivered on day 7 did not differ between RGD- and RGD++ donor hydrogels. In contrast, the number of cells delivered on day 14 using an RGD- donor hydrogel was significantly higher than the number delivered using an RGD++ donor hydrogel.

### Trilineage differentiation potential of delivered cells

**Fig 9:**
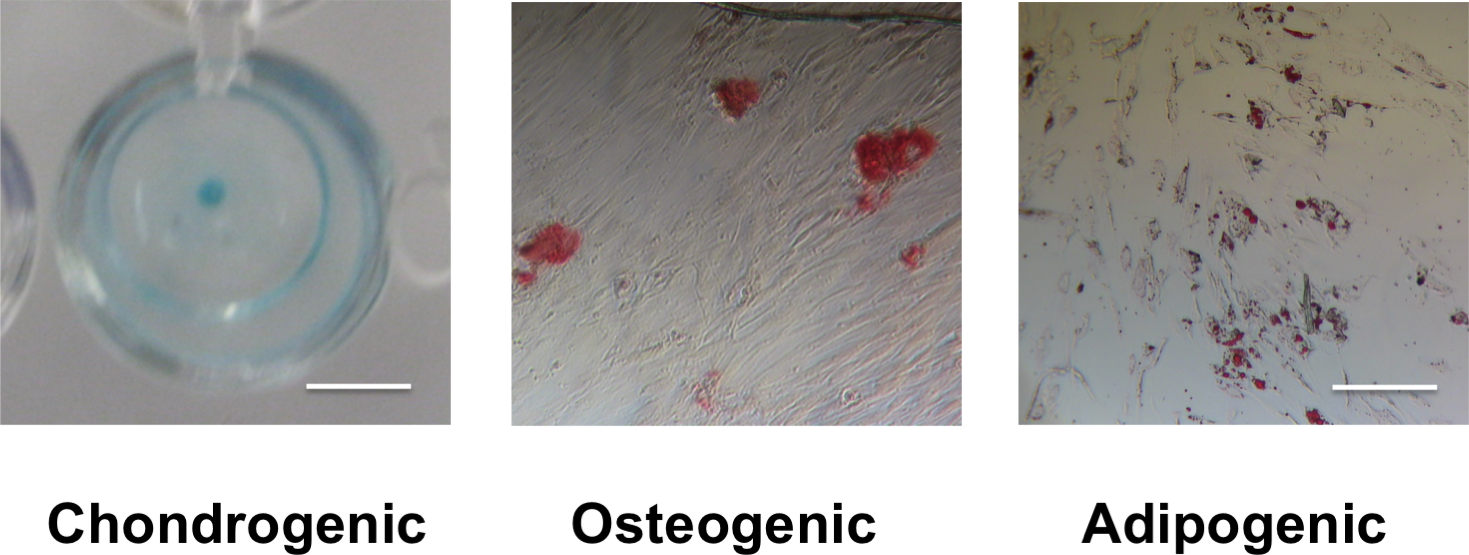
Delivered cells demonstrate trilineage differentiation potential. Staining of cells delivered from RGD- hydrogels to RGD++ hydrogels after 7 days. Delivered cells were cultured in their respective differentiation media for 21 days and stained. Delivered cells cultured in chondrogenic, osteogenic, or adipogenic differentiation medium showed production of molecules associated with chondrogenic (glycosaminoglycans, dark blue), osteogenic (calcium deposits, red), or adipogenic (lipids, red) differentiation. The scale bar in the chondrogenic images is 6.5 mm. The scale bar in the osteogenic and adipogenic images is 40 μm.

Cell delivered from RGD- hydrogels to RGD++ hydrogels after 7 days were trypsinized and tested for trilineage differentiation potential. Pellets derived from the delivered cells that were cultured in chondrogenic differentiation medium for 21 days produced a dark blue color under Alcian Blue staining, indicating the presence of glycosamioglycans (Fig 9). Delivered cells that were cultured in osteogenic differentiation medium produced a dark red color under Alizarin Red staining, indicating the presence of calcium deposits (Fig 9). However, the staining of the delivered cells was much less intense and prevalent than the staining seen with the original MSC population which suggests lower levels of calcium deposits. Delivered cells that were cultured in adipogenic medium for 21 days produced areas of red staining under Oil Red O, indicating the presence of lipids (Fig 9).

### Attachment to laminated hydrogels

**Fig 10:**
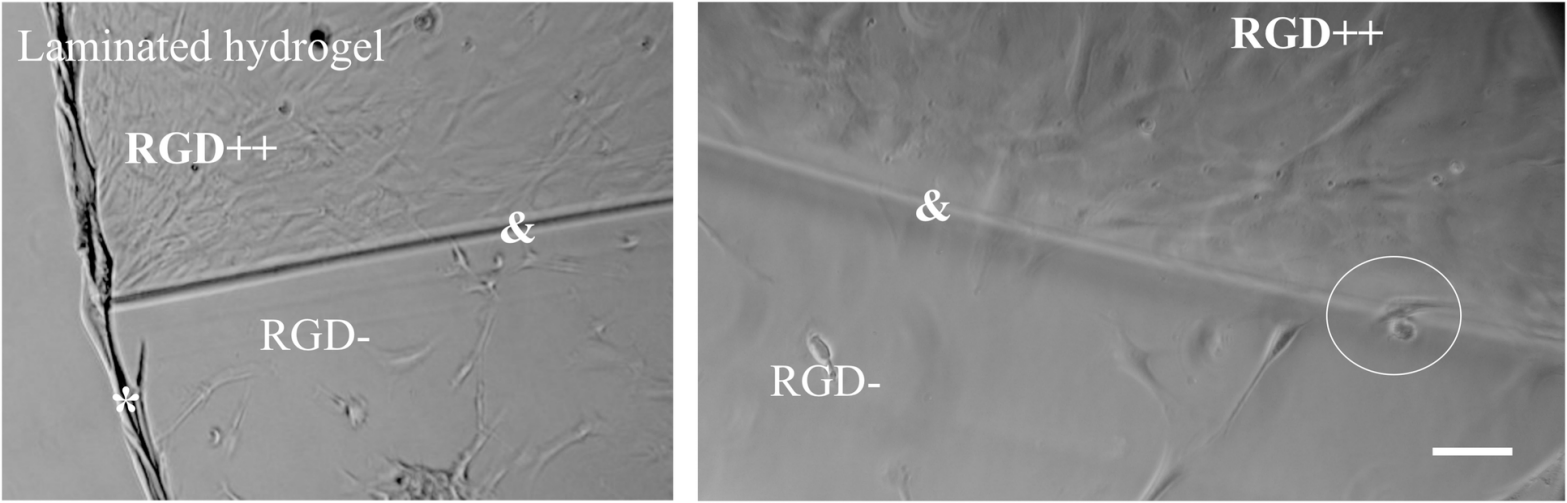
Adhesion to laminated RGD++/RGD- hydrogels. (*) indicates the edge of the laminated hydrogel. (&) Indicates the seam connecting sides of the hydrogel without RGD (RGD-) and with RGD (RGD++). The circle indicates an area where a cell is adhered across the seam. Scale bar = 10 μm.

After cross-linking was complete, there was a visually detectable seam between the RGD++/RGD- sides of the laminated hydrogels, but this seam was not perceptible through tactile examination. MSCs demonstrated the ability to adhere across the seam connecting the two sides. MSCs seeded on laminated hydrogels appeared to be present in greater numbers on the RGD++ side compared to the RGD- side by day 3 (Fig 10). This difference was especially apparent at junctions where the RGD++ and the RGD- hydrogels were joined.

## Discussion

In this study, we identified unexpected changes in cellular adhesion to RGD- PEG-DMA hydrogels. We then harnessed the particular adhesive properties of PEG-DMA hydrogels to develop a novel “bandage” delivery model to supply stem cells to a model wound site.

### Cellular attachment and release studies

In the study of cellular attachment to PEG-DA hydrogels, we investigated the relative levels of attachment to RGD- PEG-DA hydrogels compared to RGD++ hydrogels. Cells on RGD- hydrogels showed a relative lack of attachment and a rounded morphology (Figs 1 and 2), similar to previous studies with PEG-DA [11] and other PEG-based polymers [20]. The appearance of rounded cells on RGD- hydrogels was apparent throughout the entire timeframe of the study, suggesting poor adhesion. In contrast, cells appeared well spread on RGD++ hydrogels throughout the entire 15 days of culture (Fig 3).

**Fig 11:**
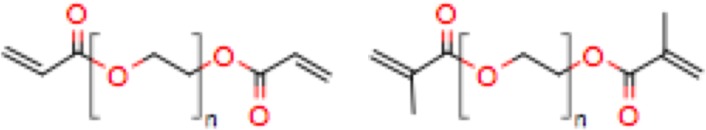
**Chemical structures of PEG-DA (left) and PEG-DMA (right).** The two chemicals only differ in structure by methyl groups near the carbon-carbon double bonds.

In the study of cellular attachment to PEG-DMA hydrogels, we examined the level of cellular attachment to RGD- PEG-DMA hydrogels and RGD++ PEG-DMA hydrogels. Since the only difference between PEG-DMA and PEG-DA is a methyl group that comprises a very small portion of the molecule (Fig 11), we expected to see results that would be virtually identical to what has been seen in similar studies with PEG-DA hydrogels. We observed that the average number of MSCs on hydrogels with high amounts of RGD (RGD++) did not significantly change over the 15 days of culture (Fig 4). In addition, images of the RGD++ hydrogels appeared to show a high cellular density with spread cells over the entire 15 day period. These results suggest MSCs can adhere to PEG-DMA hydrogels containing RGD and the adhesion is relatively stable. These results with RGD++ PEG-DMA hydrogels matched well with similar studies of PEG-based hydrogels containing RGD, including our experiment with PEG-DA hydrogels (Figs 1 and 2) and studies with PEG-DA [11] and an OPF/PEG-DA mix [20].

In studies with PEG-DA, cells do not effectively adhere to hydrogels without RGD and cells that are observed are unable to take on a spread morphology characteristic of fibroblasts and MSCs [11]. We expected to see a similar phenomenon on PEG-DMA hydrogels without RGD (RGD-). Surprisingly, fully spread MSCs were observed on RGD- hydrogels by day 3 (Fig 5), and the number of cells was not significantly different from day 1 to day 13 (Figs 4 and 5). The ability of cells to spread and adhere has also been seen in a repeated version of this experiment (S1 and S2 Figs). A recent study that grew HaCaT and MG-63 cells on PEG-DMA hydrogels for 48 hours observed cellular adhesion, spreading, and good viability [12]. Another study using L929 mouse fibroblasts on glass slides coated with PEG-DMA for three days showed some adhesion and good viability [21]. Both of these studies provide further confirmation of our result.

On day 15 the number of cells on RGD- hydrogels was significantly lower compared to day 9. On day 15 we were unable to locate any spread cells through microscopic examination (Figs 3 and 4). One potential explanation of the decreasing cell number is that MSCs can initially adhere to RGD- hydrogels, but will release from the hydrogel over time. We initially believed that the observation of cell adhesion and spreading on RGD- hydrogels was due to an experimental error, but this finding (and the decrease in cell number over time) has been observed in other iterations of this experiment (S1 and S2 Figs) and has been qualitatively observed by multiple independent researchers in our lab. In addition, when we created a laminated hydrogel with conjoined RGD- and RGD++ portions there was an observable difference in the apparent cellular density on the RGD- side compared to the RGD++ side (Fig 10). While the ability of MSCs to adhere across the seams of the laminated hydrogels and the lack of perceptible tactile differences across the junction suggests that the two sides were well-integrated, similar to previous studies [22,23], it can not be determined from this data whether the cell migrated to that location or was just initially attached at that position. The integration of the RGD-/RGD++ sides of the laminated hydrogels provides further support that cells can adhere to and spread on both RGD- and RGD++ PEG-DMA hydrogels and that differences in cell number between the two sides are not due to variations in cell populations or seeding protocols. The apparent differences in cellular density between the RGD- and RGD++ sides of the hydrogel could be explained by differences in the adhesion of MSCs to each side due to the different RGD contents in each area. The adhesive differences could be differences in the initial adhesion of MSCs to the hydrogels or differences that only manifested at a later time. Both of these interpretations would be consistent with our quantitative results (Figs 4 and 5). An alternative explanation is that there was a preferential migration of cells from one side of the laminated hydrogels to the other. It is also possible that differential attachment and preferential migration are both involved in the apparent differences in cellular density that were observed. Future quantitative studies with laminated hydrogels would be helpful to further confirm these findings and to determine the relative influences of differential attachment and/or migration on these hydrogels.

The adhesion of MSCs to PEG-DMA hydrogels was surprising because cells do not adhere well to PEG-DA hydrogels [11] (see also our data in Figs 1 and 2) which are almost chemically identical. This attachment was also surprising because PEG-DMA does not contain biological motifs (such as RGD) that would be expected to promote receptor-mediated adhesion [1]. However, it has been noted that adhesion to biomaterials can occur via attachment to proteins incidentally adsorbed to the biomaterial surface [24]. In addition, despite the high degree of chemical similarity between PEG-DA and PEG-DMA, differences in adhesion are not inconceivable, as it is known that small chemical changes can affect the presentation of adsorbed proteins thus affecting the biomaterial adhesion properties [25]. It is possible that the methyl group present in PEG-DMA promoted greater protein adsorption or a more favorable configuration for cellular adhesion compared to PEG-DA. This would explain the initial adhesion observed with RGD- PEG-DMA hydrogels. As cells proliferate on RGD- hydrogels the adsorbed proteins would need to support a greater density of cells on the hydrogel surface. Due to the lack of covalent attachment, the ability of the adsorbed proteins to anchor cells to the hydrogel may be exceeded as cells increase in density. This loss of anchoring could promote the release of cells from the RGD- surface once a certain density is reached. Such a phenomenon would explain the apparent decrease in cell number on RGD- hydrogels after an initial increase.

The apparent fall in cell number on RGD- hydrogels could also potentially be explained by an initial cell growth followed by cellular death. Perhaps cells are initially able to adhere to the RGD- hydrogels and grow, resulting in increasing cell numbers, but a lack of biological signaling motifs causes them to die over time, thus resulting in a decrease in cell number at later time points. If this were the case it would be expected that cells would initially appear healthy, but signs of cellular distress would become apparent over time as cells died off. In our study, cells of RGD- PEG-DMA hydrogels were initially observed to have a rounded morphology on day 1, but appeared well spread from day 3 to day 13 with the rounded morphology returning at day 15 (Fig 5). While the rounded morphology seen on day 1 could be a sign of weak adhesion or poor cellular health, the spread morphology seen from day 3 to 13 and the lack of changes in cell number over that timeframe are hard to explain if the cells are dying over time due to a lack of biological signaling motifs. Therefore it seems unlikely that cellular death explains the decrease in cell number at later time points. Further study examining the surface chemistry of adsorbed proteins, the strength and mechanism of cellular adhesion, and the viability of attached cells over time would be helpful in elucidating the precise mechanism of the transient changes in cell numbers that we observed.

### Delivery to strong acceptor hydrogels

The ostensible release of cells from the RGD- surfaces suggested that RGD- hydrogels could be useful as a novel tool for stem cell delivery. We hypothesized that if the RGD- hydrogels allow cells to weakly adhere, proliferate, then release over time; they may be utilized in an RGD- hydrogel as a patch covered in stem cells that could then be placed over a wound site. The cells would initially adhere to the patch and would be prevented from leaving the wound due to the RGD- surface covering the wound. Cells may then be able to leave the surface of the RGD- hydrogel and thus be delivered to the wound site. We refer to this delivery model as a “stem cell bandage”. To test this hypothesis, we applied RGD- and RGD++ donor hydrogels to an RGD++ acceptor surface.

MSCs were successfully delivered from RGD- donor hydrogels to RGD++ acceptor surfaces (Fig 6) which suggests that our “stem cell bandage” could be used to deliver cells to a surface that contains RGD adhesive peptides. The number of cells delivered from the RGD- donors to the RGD++ acceptors was not significantly different over the 21 days of the experiment. While this shows that the “stem cell bandage” can deliver cells to a model surface, it is unclear whether the cellular delivery is due to a release of cells from the donor surface, a proliferation of cells on the acceptor surface, or some other mechanism.

We did not observe a significant difference in the ability to deliver cells when an RGD++ donor hydrogel was used instead of an RGD- donor. The absence of changes between hydrogels suggests that RGD- hydrogels are not the only type capable of delivering stem cells and that RGD++ hydrogels could also be used in a “stem cell bandage” approach. The similarity between RGD- and RGD++ donor hydrogels was unexpected. We hypothesized that the lack of difference may be due to the strength of the acceptor surface. The RGD++ acceptor hydrogel, which contained high amounts of RGD, may have been so adhesive that cells strongly adhered to it no matter the adhesive properties of the donor surface. Conceptually, we think this phenomenon may be analogous to strong and weak electron acceptors in chemistry. A strong electron acceptor (i.e. with a high electronegativity) will attract electrons from both strong and weak electron donors. Similarly, our RGD++ surfaces (strong acceptor surfaces), may attract cells at similar levels irrespective of the ability of the donor surface to deliver cells. We believed that the donor surface may become more important if the acceptor surface is only weakly adhesive. In the “delivery to weak acceptor hydrogels” experiment we sought to test this hypothesis by using a relatively weak acceptor surface (RGD+).

### Delivery to weak acceptor hydrogels

RGD++ and RGD- donor hydrogels with attached cells were placed on RGD+ acceptor hydrogels that contained a relatively low concentration of RGD. This experiment was identical to the “delivery to strong acceptor hydrogels” experiment except the acceptor hydrogels used in this experiment were RGD+ hydrogels that contain a lower concentration of RGD (0.1 μmol RGD vs. 1 μmol RGD). The RGD+ acceptor hydrogels were expected to be weakly adhesive and thus to be relatively weak as cellular acceptors compared to the RGD++ hydrogels. The number of cells delivered to the RGD+ acceptor hydrogels was significantly higher on day 14 compared to day 7 for both the RGD++ and RGD- donor hydrogels, suggesting that both hydrogel donors can be successful in delivering increasing numbers of cells over time to a weakly adhesive surface.

The RGD+ acceptor hydrogels are crosslinked with fewer adhesive molecules than RGD++ donor hydrogels, but more adhesive molecules than the RGD- hydrogels. Therefore, it was believed that the RGD++ donors might retain more cells and thus deliver fewer cells than the RGD- donors. Indeed, RGD- hydrogels delivered significantly more cells on day 14 than RGD++ hydrogels, matching our prediction. This result suggests that when there is a weakly adhesive acceptor surface, such as the RGD+ acceptors, a donor hydrogel with even weaker cellular adhesion (i.e. one that strongly donates cells) such as the RGD- hydrogels, might be the best option to maximize cellular delivery. In other words, if the adhesive nature of a wound site is known it could be possible to control the dose of cells in our system by tailoring the adhesiveness of the donor hydrogel. It is outside the scope of this paper to determine the adhesive nature of particular wound sites or to create a detailed experimental plan for investigating this adhesive nature. However, some possible experimental approaches might include: a) the creation of different model wound site in an animal model followed by excision of the tissue and measurement of the RGD content, b) addition of cells to a model wound site and measurement of cellular attachment over time, or c) placement of one of our cellular bandages on a model wound site followed by quantification of cells that stay at the site over time. Further studies that characterize the adhesive nature of different types of wound sites could be valuable in improving the utility of this technology.

### Trilineage differentiation potential of delivered cells

Cells delivered from RGD- hydrogels to RGD++ hydrogels after 7 days demonstrated the ability to produce gylcosaminoglycans, calcium deposits, and lipids in response to chondrogenic, osteogenic, and adipogenic differentiation media respectively. This production suggests that these cells retain the trilineage differentiation potential (Fig 9) that was seen the initial MSCs (Fig 1). However, the osteogenic staining of the delivered cells was much less intense and prevalent than the osteogenic staining seen with the original MSC population which suggests that the delivered cells may be less effective at osteogenic differentiation and thus the trilineage differentiation potential may be lower. Given the apparently lower osteogenic potential of delivered cells, this tissue engineering strategy may be less useful for applications targeted toward bone regeneration. On the other hand, lower osteogenic differentiation might make this approach more readily applicable to applications targeted toward cartilage repair. Further investigation of differentiation strategies could improve the potential of this approach as a tissue engineering.

## Conclusion

To our knowledge, this is the first study to investigate the transient cell adhesion pattern on PEG-DMA hydrogels that would be unexpected according to data in previous studies with similar materials. In addition, we provided proof of principle for use of this transient adhesion pattern in a novel “stem cell bandage” approach to delivering cells to a wound site. We also used a model system to demonstrate how the adhesiveness of the wound site (modeled by our acceptor surfaces) and the adhesiveness of the “stem cell bandage” (modeled by our donor hydrogels) may interact to modulate the dose of cells delivered to the wound site. We were able to deliver cells to model sites with both high and low adhesive levels with increasing numbers of MSCs delivered over time.

This “bandage” approach has an advantage over direct stem cell injections as our approach prevents cells from diffusing away from the wound site, potentially increasing potency. Unlike encapsulating cells inside of a hydrogel, the “bandage” approach does not trap cells and allows access to the wound site. It may be possible to promote cellular release after encapsulation through methods such as incorporation of enzymatically degradable sequences [26] or dithiothreitol [27]. However, encapsulated cells may be prevented from proliferating and even decrease in cell number while trapped in the hydrogels [22,26]. In addition, hydrogels with high degradation rates may quickly release their cells without holding them at the wound site, thus decreasing long term cellular dose and acting similar to an injection of stem cells. On the other hand, hydrogels with low degradation rates may be able to deliver cells over time and confine the cells to the wound site, but the cellular dose in early weeks may be decreased [27]. Our system has the potential to provide a high cellular dose at early time points and maintain it over time. In addition, our system would require fewer components than encapsulation with degradable sequences, thus making it simpler, and would require fewer cells since the cells are only on the surface of our “bandage” instead of distributed throughout the hydrogel. Studies comparing our system to other systems could be valuable in revealing relative doses of cells that can be delivered to a wound site.

While the “stem cell bandage” developed in this work shows great potential as a method to deliver cells to a wound site, further work investigating the specific mechanism behind the stem cell delivery or experiments that employ this technology in an *in vivo* setting would be valuable in moving this work forward.

## Materials and methods

### Stem cell isolation

Human adipose derived MSCs were obtained through an abdominal liposuction procedure (Trinity Sports Medicine), and isolated according to previously published methods [28]. Briefly, MSCs were isolated from liposuction aspirates harvested from subcutaneous adipose tissue sites of subjects undergoing orthopedic procedures at the Trinity Sports Medicine and Performance Center Clinic. Written, informed consent was obtained from patients for this cell isolation. The research protocol used was approved by the Franciscan University of Steubenville Institutional Review Board. To isolate the MSCs, lipoaspirate samples were washed repeatedly in a syringe using Hanks Balanced Salt Solution (HBSS; Corning). After washing, adipose tissue was digested with 0.1% collagenase (type I; Worthington) in a 37°C water bath for 1 hr with gentle agitation. The digest was then centrifuged for 5 minutes at 500g to pellet the stromal vascular fraction (SVF). The SVF was resuspended in HBSS and passed through a 40 micron filter. The SVF was re-pelleted by centrifuging for 5 minutes at 500g. The cells were resuspended in appropriate growth media and the live nucleated cells were counted on a Cellometer Vision CBA cell counter (Nexcelom Bioscience) using an AO/PI dye. The isolated stromal cells were then cultured in 89% Dulbecco’s Modification Of Eagle’s Medium/ Ham’s F-12 50/50 mix with L-glutamine & 15mM HEPES (DMEM/F-12; Atlanta Biologicals), 10% Fetal bovine serum (FBS; Atlanta Biologicals), and 1% penicillin streptomycin solution (Pen/Strep; Corning), and incubated at 25°C and 5% CO_2_. This medium formulation was our basal medium.

### Trilineage differentiation potential

To test the trilineage differentiation potential of the isolated cells, the cells were grown in 6-well plates under basal medium and passaged when cells appeared approximately 80% confluent until they reached passage 4 (p4). p4 cells were tryspinized (0.25% trypsin 2.2 mM EDTA; Corning) to p5, then counted using the Cellometer Vision CBA cell counter. Portions of the cells were then deposited into new well plates for chondrogenic, osteogenic, or adipogenic differentiation (n = 2).

For chondrogenic differentiation cells were suspended at one million cells/mL. 200 μl of this solution was added to wells of a 96-well plate for a total of 200,000 cells/well. To create a pellet to facilitate chondrogenic differentiation, the plate was centrifuged at 2000 rpm for 5 minutes. After centrifugation, the plate was incubated overnight at 25°C and 5% CO_2_. The next day, chondrogenic differentiation medium was added to the cells and was changed every two days. The differentiation medium consisted of the StemPro^TM^ Chondrogenesis Differentiation Kit (ThermoFisher Scientific) and 1% penicillin streptomycin (Hyclone). Undifferentiated control cells were plated at 15,000 cells/well in 12-well plates and cultured in basal medium. After three weeks of culture the cells were stained with Alcian Blue according to previously established protocols [29] to test for evidence of chondrogenic differentiation through observation of glycosaminogylcans. Briefly, cells were washed twice in HBSS. HBSS was aspirated and a 10% formalin solution (BDH) was added for 10 minutes to fix the cells. After fixation, the formalin was aspirated and removed by washing in HBSS three times. After washing, an Alcian Blue solution was filtered through a 0.22 μm syringe filter (Corning) and added to the cells. The Alcian Blue stock consisted of a solution of 60% EtOH 40% acetic acid (EMD) with 1 mg of Alcian Blue (Alfa Aesar) per mL. The plate was covered in aluminum foil to protect it from light and incubated for 45 minutes at room temperature. After incubation the Alcian Blue solution was aspirated and destaining solution was added for 10 minutes. The destaining solution consisted of 60% EtOH and 40% acetic acid. This destaining was repeated twice. After destaining, PBS was added and the cells were imaged using a Canon PowerShot A490 camera (Canon). Dark blue staining was seen as an indication of the presence of glycosaminoglycans and thus evidence of chondrogenic differentiation.

For osteogenic differentiation cells were deposited into 12-well plates at 15,000 cells/well and incubated at 25°C and 5% CO_2_. After two days osteogenic differentiation medium was added to the cells and was changed every three days. The differentiation medium consisted of the StemPro^TM^ Osteogenesis Differentiation Kit (ThermoFisher Scientific) and 1% penicillin streptomycin. Undifferentiated control cells were plated at 15,000 cells/well in 12-well plates and cultured in basal medium. After three weeks of culture, the cells were stained with Alizarin Red according to previously established protocols [30] to test for evidence of osteogenic differentiation through observation of calcium deposits. Briefly, cells were washed twice in HBSS. HBSS was aspirated and a 10% formalin solution was added for 10 minutes to fix the cells. After fixation, the formalin was aspirated and the well was washed in HBSS three times. After washing, a 2% Alizarin Red solution (ScienCell) was added to the cells. The plate was incubated for 30 minutes at room temperature. After incubation the Alizarin Red solution was aspirated and the wells were washed with HBSS three times. After washing, the cells were imaged using a Leica Type 090-135-002 microscope. Dark red staining was seen as an indication of the presence of calcium deposits and thus evidence of osteogenic differentiation.

For adipogenic differentiation, cells were deposited into 12-well plates at 15,000 cells/well and incubated at 25°C and 5% CO_2_. After two days adipogenic differentiation medium was added to the cells and was changed every three days. The differentiation medium consisted of the StemPro^TM^ Adipogenesis Differentiation Kit (ThermoFisher Scientific) and 1% penicillin streptomycin. Undifferentiated control cells were plated at 15,000 cells/well in 12-well plates and cultured in basal medium. After three weeks of culture the cells were stained with Oil Red O according to previously established protocols [31] to test for evidence of adipogenic differentiation through observation of lipid deposits. Briefly, cells were washed twice in HBSS. HBSS was aspirated and a 10% formalin solution was added for 15 minutes to fix the cells. After fixation, the formalin was aspirated and the well was washed in HBSS three times. After washing, the Oil Red O working solution was filtered through a 0.22 μm syringe filter and added to the cells. The Oil Red O working solution consisted of 60% Oil Red O stock solution (ScienCell) and 40% deionized water. The plate was incubated for 15 minutes at room temperature. After incubation the Oil Red O solution was aspirated and the wells were washed with HBSS five times. After washing, the cells were imaged using a Leica Type 090-135-002 microscope. Dark red staining was seen as an indication of the presence of lipids and thus evidence of adipogenic differentiation.

### Poly(ethylene glycol)-dimethacrylate synthesis

PEG-DMA was created according to previous methods [9]. Briefly, poly(ethylene glycol) (PEG, nominal molecular weight 3350 Da; Sigma-Aldrich) was mixed in glass vials (max 1g/vial) with an excess of methacrylic anhydride (MA; ThermoFisher Scientific) in a 10:1 molar ratio of MA:PEG). The vials were placed in a commercial domestic microwave (Whirlpool Model No. MT2100xyr-0 Manufactured June 1992, 800W). The mixture was heated for 30s, 1min (4x), and 30s. Between heating intervals, the solution was allowed to cool as needed to prevent heat damage to the vials from the elevated temperatures. After the reaction was completed, the product was cooled to room temperature, 10 mL of anhydrous ethyl ether (EMD) was added, and the bottom of the vial was vigorously scraped to dislodge cooled product. The product was washed in 10 mL of anhydrous ethyl ether (2x) to ensure the removal of unreacted MA and isolated via Buchner filtration. Remaining solvent was removed by rotovaporation overnight and the product was stored in a -20°C freezer.

### GRGDS acrylation

To provide adhesive motifs to the hydrogels for the cells, H-Gly-Arg-Gly-Asp-Ser-OH (RGD; Calbiochem) peptide was reacted with the acrylated-PEG-succinimidyl valerate (A-PEG-SVA, MW 3400; LaysanBio, Inc.) spacer in a 1:2 molar ratio according to previous methods [20]. Briefly, GRGDS and A-PEG-SVA were combined in a sodium bicarbonate buffer (pH 8.1–8.3; ThermoFisher Scientific), under stirring, at room temperature, for 2.5 h. The mixture was dialyzed in deionized water (diH_2_O) overnight (2x) using a dialysis membrane (molecular weight cutoff of 1000 Da; Spectrum Lab) to remove any unreacted peptide. The dialyzed polymer solution was rotovapped for 24h and stored at -80°C until use.

### PEG-DA and PEG-DMA swelling study

PEG-DA (MW 4 kDa) was obtained from Polysciences, Inc. To characterize the swelling properties of PEG-DA and PEG-DMA hydrogels, RGD- PEG-DA and PEG-DMA hydrogels were cross-linked between glass slides using the thermal radical initiators ammonium persulfate (APS, 0.3M; Amresco) and N,N,N',N'-tetramethylethylenediamine (TEMED, 0.3M; ThermoFisher Scientific) for 10 min at 37°C, according to procedures established with similar PEG-based polymers [22,32,33]. Cross-linking occurred between glass slides separated by 500μm, resulting in a thin hydrogel sheet. After crosslinking, the hydrogels were cut into discs (1.63cm dia) with a cork borer. Each hydrogel disc was weighed to determine the initial crosslinking weight. The hydrogels were then swollen in deionized (DI) water overnight. After swelling, the hydrogels were blotted using weigh paper to remove excess water and weighed again to obtain a swollen weight. The swollen weight was divided by the crosslinking weight to determine the degree of hydrogel swelling after crosslinking. It was determined that PEG-DA swelled to 1.62±0.24 times the crosslinking weight (n = 11) and PEG-DMA swelled to 1.42±0.06 times the crosslinking weight (n = 12). To better account for the differences in swelling of different hydrogel types, the RGD added to the hydrogels was multiplied by these swelling ratios. In this way the ‘RGD added/swollen weight’ would be the same even for hydrogels with different swelling ratios.

### PEG-DA cell adhesion study

To characterize cellular adhesion to PEG-DA hydrogels over time, RGD++ PEG-DA hydrogels (75wt% diH_2_O) with 1μmol acrylated RGD/g swollen hydrogel or RGD- PEG-DA hydrogels were cross-linked between glass slides using the thermal radical initiators APS (0.3M) and TEMED (0.3M) for 10 min at 37°C, according to the procedures noted in the swelling study above. After crosslinking, the hydrogels were swollen in deionized (DI) water overnight and cut into discs (1.63cm dia) with a cork borer before use. Under sterile conditions, hydrogel discs (1.63cm dia) were soaked with 70% EtOH and washed twice in HBSS (10 min per wash) to remove residual EtOH. MSCs were seeded (d0) at a density of 21.0e3cells/cm^2^ on top of RGD- and RGD++ donor hydrogels and allowed to attach overnight. At each time point, cells on the hydrogels were imaged and counted. Imaging was performed using a Leica Type 090-135-002 microscope (U100/115230V~50-60HZ, Leica Microscosystems, Wetzlar, Germany GmbH). To count cells, hydrogels were first moved to a new plate in order to remove the effect of non-adherent cells and cells that may have detached from the hydrogel and attached to the bottom of the well plate. The hydrogels were then washed twice in HBSS and attached cells were trypsinized, then counted using the Cellometer Vision CBA cell counter (n = 6) on days 1 (one day after seeding), 6, 11, and 15.

### PEG-DMA cell release study

To characterize the adhesion of MSCs to PEG-DMA hydrogels over time, RGD++ PEG-DMA hydrogels (MW 3.4 kDa, 75wt% diH_2_O) with 1μmol acrylated RGD/g swollen hydrogel or RGD- hydrogels were cross-linked between glass slides using the thermal radical initiators APS (0.3M) and TEMED (0.3M) for 10 min at 37°C, according to procedures noted in the swelling study above. After crosslinking, the hydrogels were swollen in deionized (DI) water overnight and cut into discs (1.63cm dia) with a cork borer before use. Under sterile conditions, hydrogel discs were soaked with 70% EtOH and washed twice in HBSS (10 min per wash) to remove residual EtOH. MSCs were seeded (d0) at a density of 21.0e3cells/cm^2^ on top of RGD- and RGD++ donor hydrogels and allowed to attach overnight. At each time point, cells on the hydrogels were imaged and counted. Imaging was performed using a Leica Type 090-135-002 microscope. To count cells, hydrogels were first moved to a new plate in order to remove the effect of non-adherent cells and cells that may have detached from the hydrogel and attached to the bottom of the well plate. The hydrogels were then washed twice in HBSS and attached cells were trypsinized (0.25% trypsin 2.2 mM EDTA), then counted using the Cellometer Vision CBA cell counter (n = 6) on days 1 (one day after seeding), 3, 6, 9, 11, 13, and 15.

### Cell delivery study to strong acceptor hydrogel

In order to determine if cells could be delivered from a hydrogel to a model surface, RGD++ and RGD- hydrogel discs (dia 1.63cm) (MW 3.4 kDa, 75wt% diH2O) were fabricated, swollen, and sterilized using the same procedures noted in the cell release study above. MSCs were seeded at a density of 21.0e3cells/cm^2^ on top of RGD- and RGD++ hydrogels and allowed to attach overnight. Since these hydrogels were being used to deliver MSCs they are referred to as “donor hydrogels”. After attachment, donor hydrogels were moved to new 12-well plates and an RGD++ hydrogel was placed on top of the donor hydrogel. The top hydrogels were used to model an adhesive target surface and were thus termed “acceptor hydrogels”. To achieve maximum contact between the hydrogels, an autoclaved stainless steel washer (0.9cm) was placed on top of each donor/acceptor complex. After 24h, 7 days, 14 days, and 21 days, acceptor hydrogels were removed and cells present on those hydrogels were trypsinized and counted (n = 6).

### Cell delivery study to weak acceptor hydrogel

In order to determine whether the RGD concentration of the acceptor hydrogel affects cell delivery, RGD++, RGD+ and RGD- hydrogels (dia 1.63cm, MW 3.4 kDa, 75wt% diH2O) were fabricated, swollen, and sterilized using the same procedures noted in the cell release study above. MSCs were seeded at a density of 21.0e3cells/cm^2^ on top of the RGD- or RGD++ donor hydrogels and allowed to attach overnight. After attachment, the donor hydrogels were moved to new 12-well plates and an RGD+ acceptor hydrogel was placed on top of the donor hydrogel. To achieve maximum contact between the gels, an autoclaved stainless steel washer (0.9cm dia) was placed on top of each donor/acceptor complex. After 24h, 7 days, and 14 days, the acceptor hydrogels were removed and cells present on the acceptor hydrogel were trypsinized and counted (n = 5).

### Trilineage differentiation potential of delivered cells

In order to determine whether stem cells delivered from PEG-DMA hydrogels maintain their trilineage differentiation potential, RGD++ and RGD- hydrogel discs (dia 1.63cm) (MW 3.4 kDa, 75wt% diH2O) were fabricated, swollen, and sterilized using the same procedures noted in the cell release study above. MSCs were seeded at a density of 21.0e3cells/cm^2^ on top of RGD- hydrogels and allowed to attach overnight. After attachment, donor hydrogels were moved to new 12-well plates and an RGD++ hydrogel was placed on top of the donor hydrogel. To achieve maximum contact between the hydrogels, an autoclaved stainless steel washer (0.9cm) was placed on top of each donor/acceptor complex. After 7 days, acceptor hydrogels were removed and cells present on those hydrogels were trypsinized, counted, and deposited into new well plates. The trilineage differentiation potential of cells from the acceptor hydrogels was tested using the methods described above in the “trilineage differentiation potential” section (n = 2).

### Laminated hydrogels

Laminated hydrogels were fabricated to create a single unified hydrogel containing an RGD++ side and an RGD- side, similar to previous methods [22,23]. Briefly, an RGD- hydrogel mixture was placed on half of a glass slide complex and allowed to cross-link for 6 min at 37°C. The RGD++ hydrogel mixture was then added to the unoccupied side of the slide complex until it bordered the semi-polymerized RGD- hydrogel. The complex was then cross-linked for another 8 minutes to complete both sides of the laminated hydrogel. MSCs were seeded at a density of 21.0e3cells/cm^2^ on top of laminated RGD-/RGD++ hydrogels, and allowed to attach overnight, then imaged.

### Statistical analysis

All data is reported as an average +/- standard deviation. Data was analyzed for statistical significance by first using a Q-test to remove statistical outliers, then by using a two-way ANOVA followed by Tukey’s post-hoc test (*p* ≤ 0.05).

## Supporting information

S1 Fig Repeated run of Fig 4. Number of cells present on RGD- and RGD++ hydrogels after 1, 11, 14 days (n = 6 ± standard deviation). ^(1)^Significance vs. day 1 for the same sample type. ^‡^Significance from RGD- hydrogels at same time point (p < 0.05).
(TIF)

S2 Fig Repeated run of Fig 5. Cells adhered to PEG-DMA hydrogels with RGD peptides (RGD++) and without peptides (RGD-) on days 1, 11, and 14. Inset: an alternate area of the hydrogel showing a different pattern of cellular adhesion. Scale bar = 10 μm.
(TIF)

S3 Fig Repeated run of Fig 6. Increasing numbers of cells are delivered by RGD- donor hydrogels (strong donor) to the RGD++ acceptor hydrogel surface over time. Cell numbers on RGD++ acceptor hydrogels were measured after 1, 7, and 14 days (n = 5 ± standard deviation). ^(1)^Significance from d1 hydrogels for the same sample type. ^(7)^Significance from d7 hydrogels for the same sample type (p < 0.05).
(TIF)

S4 Fig Fig 2 raw data. (XLSX)

S5 Fig Fig 4 raw data. (XLSX)

S6 Fig Fig 6 raw data. (XLSX)

S7 Fig Fig 8 raw data. (XLSX)

S8 Fig S1 Fig raw data. (XLSX)

S9 Fig S2 Fig raw data. (XLSX)

S10 Fig Raw data for PEG-DA and PEG-DMA swelling study. (XLSX)
