# Supplementary figures and images for: Transient cellular adhesion on poly(ethylene-glycol)-dimethacrylate hydrogels facilitates a novel stem cell bandage approach
Source: PLoS One. 2018 Aug 23;13(8):e0202825. doi: 10.1371/journal.pone.0202825 (PMC6107244; doi:10.1371/journal.pone.0202825)

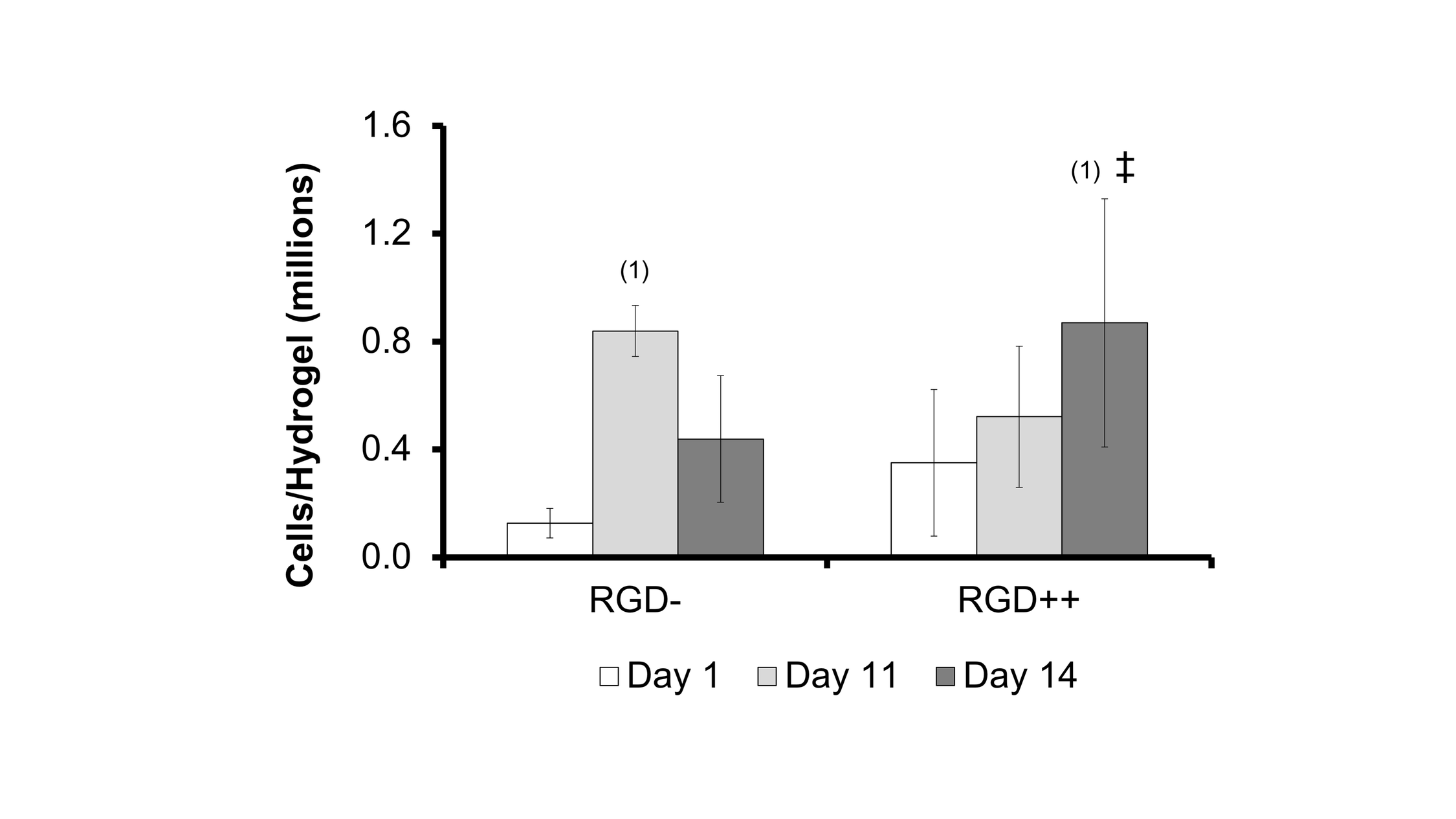

Supplement: S1 Fig — Number of cells present on RGD- and RGD++ hydrogels after 1, 11, 14 days (n = 6 ± standard deviation). (1)Significance vs. day 1 for the same sample type. ‡Significance from RGD- hydrogels at same time point (p < 0.05). (TIF) [file pone.0202825.s001.tif]

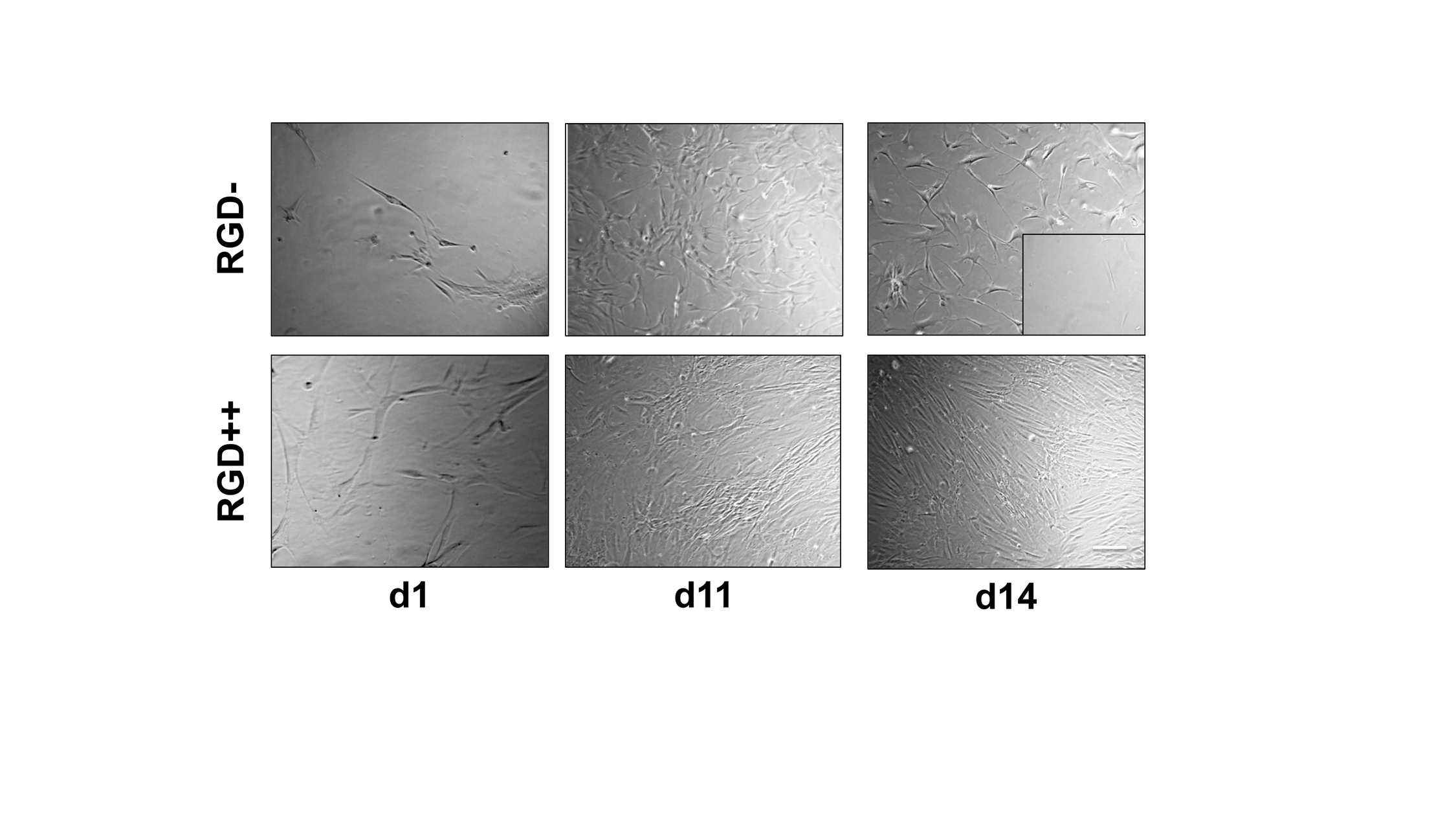

Supplement: S2 Fig — Cells adhered to PEG-DMA hydrogels with RGD peptides (RGD++) and without peptides (RGD-) on days 1, 11, and 14. Inset: an alternate area of the hydrogel showing a different pattern of cellular adhesion. Scale bar = 10 μm. (TIF) [file pone.0202825.s002.tif]

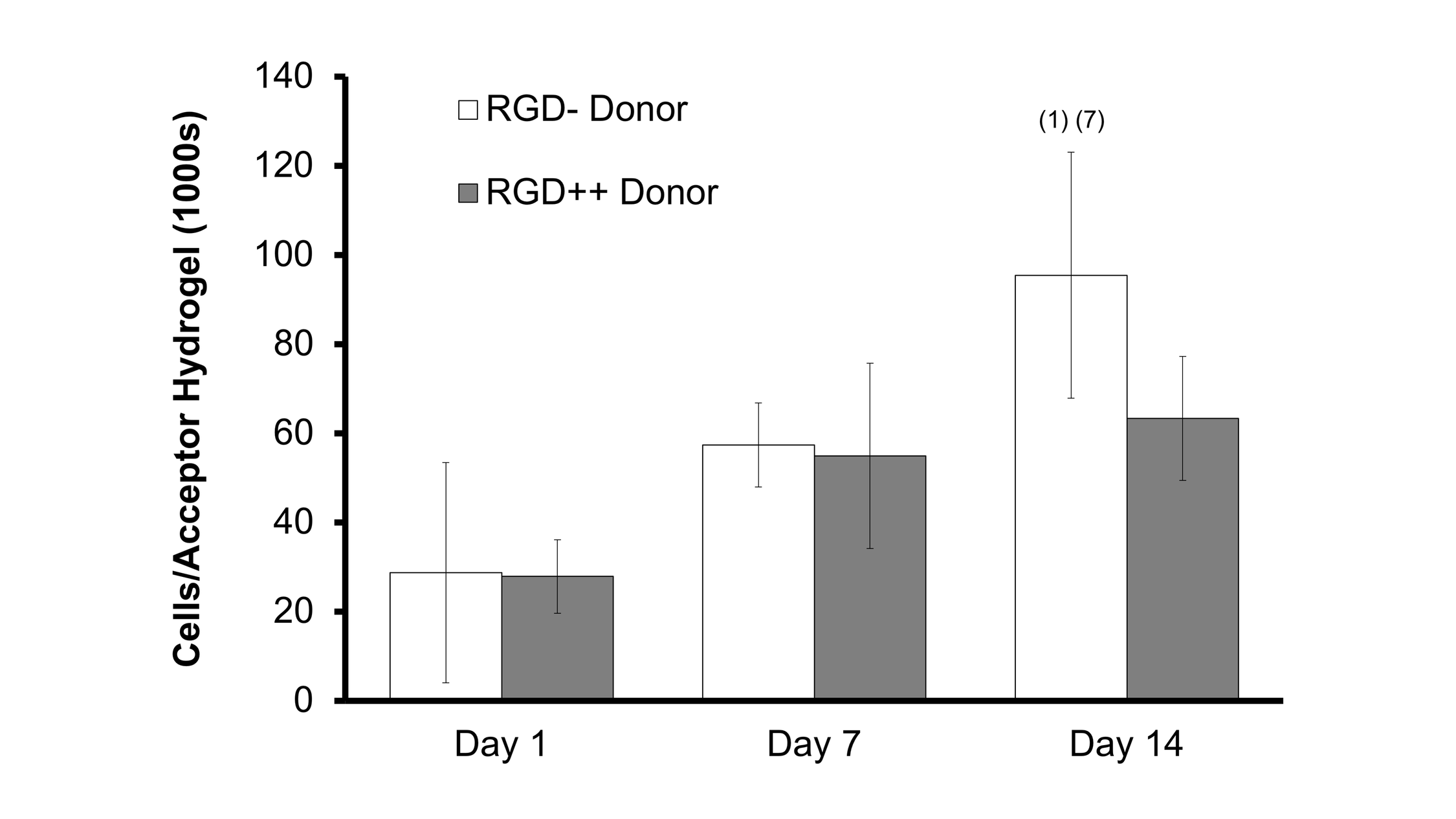

Supplement: S3 Fig — Increasing numbers of cells are delivered by RGD- donor hydrogels (strong donor) to the RGD++ acceptor hydrogel surface over time. Cell numbers on RGD++ acceptor hydrogels were measured after 1, 7, and 14 days (n = 5 ± standard deviation). (1)Significance from d1 hydrogels for the same sample type. (7)Significance from d7 hydrogels for the same sample type (p < 0.05). (TIF) [file pone.0202825.s003.tif]
